# Association of Brain Atrophy With Disease Progression Independent of Relapse Activity in Patients With Relapsing Multiple Sclerosis

**DOI:** 10.1001/jamaneurol.2022.1025

**Published:** 2022-05-16

**Authors:** Alessandro Cagol, Sabine Schaedelin, Muhamed Barakovic, Pascal Benkert, Ramona-Alexandra Todea, Reza Rahmanzadeh, Riccardo Galbusera, Po-Jui Lu, Matthias Weigel, Lester Melie-Garcia, Esther Ruberte, Nina Siebenborn, Marco Battaglini, Ernst-Wilhelm Radue, Özgür Yaldizli, Johanna Oechtering, Tim Sinnecker, Johannes Lorscheider, Bettina Fischer-Barnicol, Stefanie Müller, Lutz Achtnichts, Jochen Vehoff, Giulio Disanto, Oliver Findling, Andrew Chan, Anke Salmen, Caroline Pot, Claire Bridel, Chiara Zecca, Tobias Derfuss, Johanna M. Lieb, Luca Remonda, Franca Wagner, Maria I. Vargas, Renaud Du Pasquier, Patrice H. Lalive, Emanuele Pravatà, Johannes Weber, Philippe C. Cattin, Claudio Gobbi, David Leppert, Ludwig Kappos, Jens Kuhle, Cristina Granziera

**Affiliations:** 1 Translational Imaging in Neurology (ThINK) Basel, Department of Biomedical Engineering, Faculty of Medicine, University Hospital Basel, University of Basel, Basel, Switzerland; 2 Neurologic Clinic and Policlinic, MS Center and Research Center for Clinical Neuroimmunology and Neuroscience Basel (RC2NB), University Hospital Basel, University of Basel, Basel, Switzerland; 3 Clinical Trial Unit, Department of Clinical Research, University Hospital Basel, University of Basel, Basel, Switzerland; 4 Division of Diagnostic and Interventional Neuroradiology, Clinic for Radiology and Nuclear Medicine, University Hospital Basel, University of Basel, Basel, Switzerland; 5 Division of Radiological Physics, Department of Radiology, University Hospital Basel, Basel, Switzerland; 6 Medical Image Analysis Center (MIAC) and Quantitative Biomedical Imaging Group, Department of Biomedical Engineering, University of Basel, Basel, Switzerland; 7 Department of Medicine, Surgery and Neuroscience, University of Siena, Siena, Italy; 8 Department of Neurology, Cantonal Hospital St. Gallen, St. Gallen, Switzerland; 9 Department of Neurology, Cantonal Hospital Aarau, Aarau, Switzerland; 10 Neurology Department, Neurocenter of Southern Switzerland, Lugano, Switzerland; 11 Department of Neurology, Inselspital, Bern University Hospital, University of Bern, Bern, Switzerland; 12 Division of Neurology, Department of Clinical Neurosciences, Lausanne University Hospital (CHUV), University of Lausanne, Lausanne, Switzerland; 13 Division of Neurology, Department of Clinical Neurosciences, Faculty of Medicine, Geneva University Hospitals, Geneva, Switzerland; 14 Faculty of Biomedical Sciences, Università della Svizzera Italiana, Lugano, Switzerland; 15 Department of Radiology, Cantonal Hospital Aarau, Aarau, Switzerland; 16 Department of Diagnostic and Interventional Neuroradiology, Inselspital, Bern University Hospital, University of Bern, Bern, Switzerland; 17 Department of Radiology, Faculty of Medicine, Geneva University Hospital, Geneva, Switzerland; 18 Division of Radiology, Lausanne University Hospital (CHUV), University of Lausanne, Lausanne, Switzerland; 19 Department of Neuroradiology, Neurocenter of Southern Switzerland, Lugano, Switzerland; 20 Department of Radiology, Cantonal Hospital St. Gallen, St. Gallen, Switzerland; 21 Center for Medical Image, Analysis, and Navigation, Department of Biomedical Engineering, University of Basel, Allschwil, Switzerland

## Abstract

**Importance:**

The mechanisms driving neurodegeneration and brain atrophy in relapsing multiple sclerosis (RMS) are not completely understood.

**Objective:**

To determine whether disability progression independent of relapse activity (PIRA) in patients with RMS is associated with accelerated brain tissue loss.

**Design, Setting, and Participants:**

In this observational, longitudinal cohort study with median (IQR) follow-up of 3.2 years (2.0-4.9), data were acquired from January 2012 to September 2019 in a consortium of tertiary university and nonuniversity referral hospitals. Patients were included if they had regular clinical follow-up and at least 2 brain magnetic resonance imaging (MRI) scans suitable for volumetric analysis. Data were analyzed between January 2020 and March 2021.

**Exposures:**

According to the clinical evolution during the entire observation, patients were classified as those presenting (1) relapse activity only, (2) PIRA episodes only, (3) mixed activity, or (4) clinical stability.

**Main Outcomes and Measures:**

Mean difference in annual percentage change (MD-APC) in brain volume/cortical thickness between groups, calculated after propensity score matching. Brain atrophy rates, and their association with the variables of interest, were explored with linear mixed-effect models.

**Results:**

Included were 1904 brain MRI scans from 516 patients with RMS (67.4% female; mean [SD] age, 41.4 [11.1] years; median [IQR] Expanded Disability Status Scale score, 2.0 [1.5-3.0]). Scans with insufficient quality were excluded (n = 19). Radiological inflammatory activity was associated with increased atrophy rates in several brain compartments, while an increased annualized relapse rate was linked to accelerated deep gray matter (GM) volume loss. When compared with clinically stable patients, patients with PIRA had an increased rate of brain volume loss (MD-APC, −0.36; 95% CI, −0.60 to −0.12; *P* = .02), mainly driven by GM loss in the cerebral cortex. Patients who were relapsing presented increased whole brain atrophy (MD-APC, −0.18; 95% CI, −0.34 to −0.02; *P* = .04) with respect to clinically stable patients, with accelerated GM loss in both cerebral cortex and deep GM. No differences in brain atrophy rates were measured between patients with PIRA and those presenting relapse activity.

**Conclusions and Relevance:**

Our study shows that patients with RMS and PIRA exhibit accelerated brain atrophy, especially in the cerebral cortex. These results point to the need to recognize the insidious manifestations of PIRA in clinical practice and to further evaluate treatment strategies for patients with PIRA in clinical trials.

## Key Points

**Question:**

Is disability progression independent of relapse activity (PIRA) in relapsing multiple sclerosis associated with increased rates and specific patterns of brain atrophy?

**Findings:**

In this cohort study that included 516 patients with relapsing multiple sclerosis and 1904 brain magnetic resonance imaging scans, PIRA was associated with significantly increased brain volume loss. With respect to clinically stable patients, patients with PIRA presented an accelerated total brain atrophy, which was particularly evident in the cerebral cortex.

**Meaning:**

The association between PIRA and brain atrophy points at the need to identify insidious disease progression in patients with relapsing multiple sclerosis and to further investigate therapeutic approaches to prevent irreversible brain tissue loss in these patients.

## Teaser

This cohort study examines magnetic resonance imaging scans to determine changes in regional and total brain volume among patients with relapsing multiple sclerosis who have disease progression without relapse activity.

## Introduction

Multiple sclerosis (MS) is a chronic disease of the central nervous system characterized by inflammatory, demyelinating, and neurodegenerative processes.^1^ Despite significant progress in the clinical management of patients with MS, the mechanisms driving disability accumulation are not fully understood.

While it is widely accepted that disability accrual may result from the neuroinflammatory events occurring in clinical relapses (relapse-associated worsening),^2^ it is much less clear why some patients experience disability progression independent of relapse activity (PIRA).

Notably, while the insidious accumulation of disability is characteristic of the progressive MS disease courses,^3^ PIRA has recently emerged as a crucial clinical feature also in relapsing MS (RMS).^4^ Indeed, PIRA has been shown to occur in typical RMS populations, challenging the traditional distinction between an early exclusively relapsing phase and a late secondary progressive MS (SPMS). The pathophysiological determinants of PIRA remain elusive, although it is plausible that PIRA is associated with increased diffuse neuroaxonal loss.

Assessment of brain atrophy by magnetic resonance imaging (MRI) enables the in vivo quantification of ongoing neurodegenerative processes. Accelerated brain tissue loss is a critical phenomenon in MS, presenting close association with clinical disability, and involving multiple central nervous system compartments with as yet poorly understood differences in regional magnitude and temporal evolution.^7^ Brain tissue loss may be the consequence of acute focal neuroinflammatory events as well as more diffuse primary or secondary neurodegenerative processes that occur independent from lesion activity.^8^ These include chronic focal “smoldering” activity,^9^ progressive loss of chronically demyelinated axons outside MS lesions, astrocyte damage and microglia activation in the normal-appearing white matter (WM) tissue,^11^ and meningeal inflammation leading to subpial gray matter (GM) pathology.^12^

In this work, we aimed at investigating whether PIRA is associated with brain volume loss and whether the pace and pattern of brain volume loss in patients with PIRA are distinct from those observed in patients with clinical relapse-associated worsening. In a large cohort of patients with RMS, we therefore assessed the association between global and regional rates of brain atrophy and (1) focal inflammatory activity (evaluated both clinically and radiologically) and (2) PIRA.

## Methods

### Participants

This longitudinal retrospective investigation included patients prospectively followed up in the Swiss Multiple Sclerosis Cohort, an observational multicentric study with standardized collection of demographics, clinical, and MRI data.^13^ The study was approved by the local ethics committee. Written informed consent was obtained from all participants before study enrollment. The study follows the Strengthening the Reporting of Observational Studies in Epidemiology (STROBE) guideline for reporting observational studies.^14^

Patients participating in the Swiss Multiple Sclerosis Cohort study were enrolled in the present study according to the following inclusion criteria: (1) availability of at least 2 brain MRIs, including 1-mm isotropic magnetization-prepared rapid gradient-echo (MPRAGE) and T2/fluid-attenuated inversion recovery (FLAIR), separated in time by at least 6 months; (2) diagnosis of RMS fulfilling the 2017 revisions of the McDonald criteria^15^; (3) availability of at least 1 annual clinical follow-up, with temporal proximity between MRI acquisition and neurological evaluation (≤2 months); and (4) age between 18 and 80 years. All clinical and MRI data acquired as part of the Swiss Multiple Sclerosis Cohort study between January 2012 and September 2019 from patients fulfilling eligibility criteria were included. MRI scans with insufficient image quality were excluded.

### Clinical Data

Demographic and clinical data included sex, age, disease duration (defined as time since first symptom), and current and previous disease-modifying therapies (DMTs). Standardized clinical assessments with Expanded Disability Status Scale (EDSS) calculation were performed by certified raters every 6 or 12 months.^13^ The occurrence of relapses (defined according to the McDonald criteria^15^) was recorded at each visit.

Confirmed disability progression was defined as an EDSS score increase, confirmed at least after 6 months, of (1) 1.5 or more points if baseline EDSS was 0, (2) 1.0 or more points if baseline EDSS was 1.0 to 5.5, or (3) 0.5 or more points if baseline EDSS was greater than 5.5. PIRA was defined as an episode of confirmed disability progression with no relapse during the 90 days before the EDSS increase and during the 6-month period between the EDSS increase and the confirmation of disability progression.

According to the longitudinal clinical evolution, we distinguished:

1. Patients with relapse activity and without PIRA: presenting at least 1 relapse (irrespective of whether it was associated with confirmed disability progression) and without PIRA events during the entire observation;
2. Patients with exclusive PIRA: presenting at least 1 event of PIRA and without relapses during the entire observation;
3. Patients with mixed activity: presenting at least 1 relapse and 1 episode of PIRA;
4. Stable patients: without relapses or PIRA during the entire observation.

**Figure 1.**
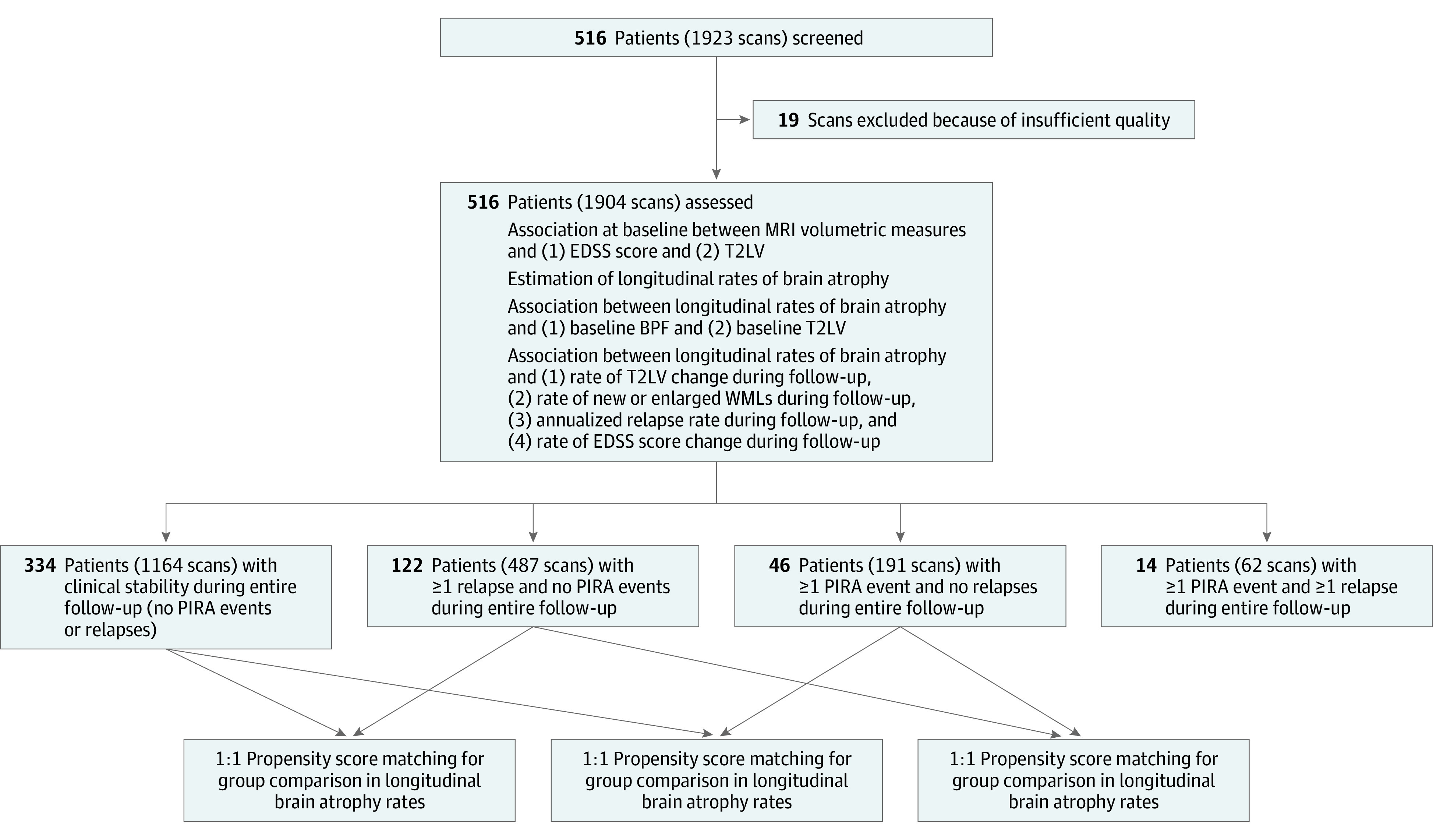
Study Design BPF indicates brain parenchymal fraction; EDSS, Expanded Disability Status Scale; PIRA, progression independent of relapse activity; T2LV, T2 lesion volume; WMLs, white matter lesions.

The study design is outlined in Figure 1.

### MRI Acquisition

Brain MRI scans were performed at each center using protocols optimized for homogeneous signal-to-noise ratio (eTable 1 in the Supplement). All protocols included a 3-dimensional (3-D), T1-weighted, 1-mm isotropic MPRAGE and a 3-D, 1-mm isotropic FLAIR.

### MRI Analysis

Brain MRI analysis was conducted in Basel, Switzerland. Before analysis all native images were visually assessed to ensure sufficient quality.

T2 lesion volume (T2LV) was calculated automatically on FLAIR images using the multidimensional gated recurrent units algorithm,^17^ and results were manually reviewed. Longitudinal changes of WM lesions were automatically assessed with LeMan-PV,^18^ and the outputs, in terms of new and enlarged lesions, were manually reviewed (A. Cagol, R.A.T., N.S.).

Volumetric analysis was performed on MPRAGE images after lesion filling.^19^ Volumetric segmentation and cortical reconstruction were obtained with the longitudinal stream of FreeSurfer (version 6.0).^20^ Results were reviewed (A. Cagol) and, if needed, manually corrected according to FreeSurfer recommendations.

Estimations for the regions of interest, including total brain volume (TBV) and volumes of total GM, total WM, cerebral cortex, deep GM, thalamus, cerebellum, and ventricular system, were obtained from FreeSurfer; for symmetric structures, left and right volumes were summed. Mean cortical thickness (CTh) was quantified for the whole cortex, as well as for each lobar and regional cortical area (according to the Desikan-Killiany atlas^25^), as the average of the thickness obtained in the 2 hemispheres. Total intracranial volume (TIV) was used as a covariate in the analyses to adjust for between-patient differences in head size. SPM12 ^26^ was preferred to FreeSurfer for measuring TIV because it provides a direct quantification instead of an indirect estimation.^27^

In this study, we chose the longitudinal pipeline of FreeSurfer to explore brain volume changes over time because (1) it gives the opportunity to consider several brain regions, including structures characteristically affected in MS pathology, and (2) it provides measures of global and regional CTh. In FreeSurfer, the inherent variability associated with the independent analysis of each time point of a patient is addressed with a dedicated longitudinal pipeline by creating an unbiased, robust, within-patient template.^24^ Such approach proved to increase sensitivity and statistical power in detecting subtle longitudinal changes.^24^

To support the reliability of brain volumetric measures in our data set, TBV and deep GM volume were quantified in all MRI scans also using SPM12 ^26^ and FIRST,^28^ respectively, and brain atrophy rates were obtained also with Structural Image Evaluation, using Normalization, of Atrophy (SIENA).^29^ The agreement between software packages is reported in the eMethods in the Supplement.

### Statistical Analysis

All statistical analysis was conducted in R version 3.6.3.^30^ Data were analyzed between January 2020 and March 2021. Linear mixed-effect models^31^ were performed using the lme4 package^32^ to do 5 types of analyses. First, we investigated the cross-sectional association at baseline between brain volumetric measurements (dependent variable) and (1) EDSS score and (2) T2 lesion load (estimated as the logarithmic transformation of T2LV). Models included TIV, sex, age, and disease duration as covariates and MRI protocol (defined by the combination of center and scanner) as random intercept.

Second, a linear mixed-effect model was used to quantify the longitudinal rates of atrophy during follow-up. Brain measurements at each given time point were used as dependent variables. To estimate annual percentage change in brain volume from the slope over time, brain measurements were log-transformed.^33^ Models included as covariates time (to estimate the rate of volume/thickness change), sex, TIV, age and disease duration at baseline, as well as the interactions between sex and baseline disease duration with time (to adjust the rate of change for sex and disease duration). In addition, both random intercepts (for participants and MRI protocols) and a random slope (on time) were included.

Third, we assessed the association between longitudinal rates of atrophy and (1) brain parenchymal fraction (calculated as the ratio between TBV and TIV) at baseline and (2) T2 lesion load at baseline. The association was investigated by introducing in the model the interaction term between time and the variables of interest.

Fourth, we explored the association between rates of volume/thickness change (dependent variable) and (1) the rate of change in lesion burden, (2) the annualized relapse rate, and (3) the rate of change in EDSS score. The association was investigated by introducing in the model the interaction term between time and the variables of interest.

Fifth, the rates of brain atrophy were compared between (1) patients with exclusive PIRA activity and stable patients; (2) patients with relapse activity but no PIRA and stable patients; and (3) patients with exclusive PIRA activity and patients with relapse activity but no PIRA. The mean difference in annual percentage volume/thickness change (MD-APC) was assessed as the interaction term between patient group and time. The 3 comparisons were performed after a 1:1 nearest-neighbor propensity score matching, including duration of follow-up, age, sex, disease duration, number of scans available, and treatment with DMTs as criteria. The balance between groups was assessed with Pearson χ^2^ and Mann-Whitney *U* tests. Comparisons of demographic, clinical, and MRI measures between groups were investigated with Welch *t* test, Pearson χ^2^ test, and Mann-Whitney *U* test as appropriate.

Results were corrected for multiple comparisons using the false discovery rate approach; reported *P* values are adjusted for false discovery rate. The threshold of statistical significance was set at *P* < .05. Graphical results for CTh analysis were displayed with the fsbrain package.^34^ To assess the reliability of brain volumetric measures obtained with different software packages, an intraclass correlation coefficient was calculated.^35^ As a sensitivity analysis, the effect of DMTs on brain atrophy rates was investigated.

## Results

A total of 1904 brain MRI scans from 516 patients were included; 19 scans had to be excluded because of insufficient quality. No patients were excluded. Median (IQR) follow-up was 3.2 (2.0-4.9) years, with a median (IQR) number of scans per patient of 4 (2-5). During the observation period, 46 patients experienced only PIRA events and no relapses, 122 patients relapse activity without PIRA, 14 patients both PIRA and relapse activity, and 334 patients remained clinically stable. The cohort’s characteristics are summarized in Table 1.

**Table 1.** Clinical and MRI Characteristics in the Entire Cohort and in the 4 Groups of Patients

	Cohort (n = 516)	PIRA (n = 46)	Relapsing (n = 122)	PIRA + Relapsing (n = 14)	Stable (n = 334)
**Demographic and clinical data**					
Female, No. (%)	348 (67)	34 (74)	94 (77)	11 (79)	209 (63)
Age at baseline, mean (SD), y	41.4 (11.1)	45.5 (10.8)	39.0 (9.8)	39.9 (13.6)	41.7 (11.2)
Disease duration at baseline, mean (SD), y	9.5 (8.1)	11.1 (9.0)	9.6 (8.2)	11.5 (6.8)	9.1 (8.0)
EDSS score at baseline, median (IQR)	2.0 (1.5 to 3.0)	2.0 (1.5 to 2.875)	2.0 (1.5 to 3.0)	2.5 (2.0 to 3.375)	2.0 (1.5 to 3.0)
Patients taking DMTs at baseline, No. (%)	423 (82)	37 (80)	103 (84)	12 (86)	271 (81)
Group 1 DMTs at baseline, No.	77	4	17	3	53
Group 2 DMTs at baseline, No.	253	27	66	7	153
Group 3 DMTs at baseline, No.	93	6	20	2	65
Follow-up duration, median (IQR), y	3.2 (2.0 to 4.9)	4.0 (2.0 to 5.0)	4.0 (3.0 to 5.0)	5.0 (3.8 to 5.6)	3.0 (1.5 to 4.3)
ARR, mean (SD)	0.12 (0.30)	0 (0)	0.48 (0.43)	0.31 (0.16)	0 (0)
Annualized ΔEDSS rate, median (IQR)	0 (0 to 0)	0.20 (0.11 to 0.50)	0.00 (−0.06 to 0.23)	0.19 (0.11 to 0.32)	0 (0 to 0)
**MRI data**					
Brain MRI scans, total No.	1904	191	487	62	1164
No. of scans per patient, median (IQR)	4 (2 to 5)	4 (3 to 5)	4 (3 to 5)	4 (4 to 5)	3 (2 to 5)
BPF at baseline, median (IQR)	0.762 (0.725 to 0.792)	0.756 (0.715 to 0.788)	0.789 (0.764 to 0.812)	0.773 (0.753 to 0.797)	0.775 (0.738 to 0.807)
T2LV at baseline, median (IQR), mL	4.0 (1.6 to 11.9)	5.7 (1.8 to 15.1)	4.5 (1.1 to 12.1)	5.8 (2.9 to 11.4)	3.8 (1.6 to 11.5)
Annualized ΔT2LV, median (IQR), mL	0.04 (−0.22 to 0.48)	−0.02 (−0.20 to 0.73)	0.10 (−0.12 to 0.85)	0.16 (−0.08 to 1.20)	0.03 (−0.28 to 0.37)
Annualized No. of new/enlarged T2 lesions, median (IQR)	0.17 (0 to 1.25)	0.20 (0 to 1.40)	0.62 (0 to 3.28)	1.21 (0.17 to 4.13)	0 (0 to 0.828)

Abbreviations: ARR, annualized relapse rate; BPF, brain parenchymal fraction; ΔEDSS, change in EDSS score; ΔT2LV, change in T2 lesion volume; EDSS, Expanded Disability Status Scale; DMTs, disease-modifying therapies; DMTs group 1: interferon beta, glatiramer-acetate; DMTs group 2: teriflunomide, dimethyl fumarate, fingolimod; DMTs group 3: natalizumab, rituximab, ocrelizumab, alemtuzumab; MRI, magnetic resonance imaging; PIRA: progression independent of relapse activity; T2LV, T2 lesion volume.

### Association of EDSS Score and T2LV With Brain Measurements at Baseline

At baseline, both EDSS score and T2LV were associated with TBV (β, −0.081; 95% CI, −0.134 to −0.028; *P* = .01, and β, −0.136; 95% CI, −0.186 to −0.087; *P* < .001, respectively), as well as with total GM (β, −0.074; 95% CI, −0.133 to −0.017; *P* = .04, and β, −0.094; 95% CI, −0.149 to −0.039; *P* = .001, respectively), and total WM (β, −0.069; 95% CI, −0.125 to −0.012; *P* = .04, and β, −0.154; 95% CI, −0.207 to −0.101; *P* < .001, respectively). The strongest regional association was detected with thalamic volume (β, −0.187; 95% CI, −0.255 to −0.120; *P* < .001, and β, −0.354; 95% CI, −0.412 to −0.295; *P* < .001, respectively) (eTable 2 in the Supplement).

### Rates of Atrophy

**Figure 2.**
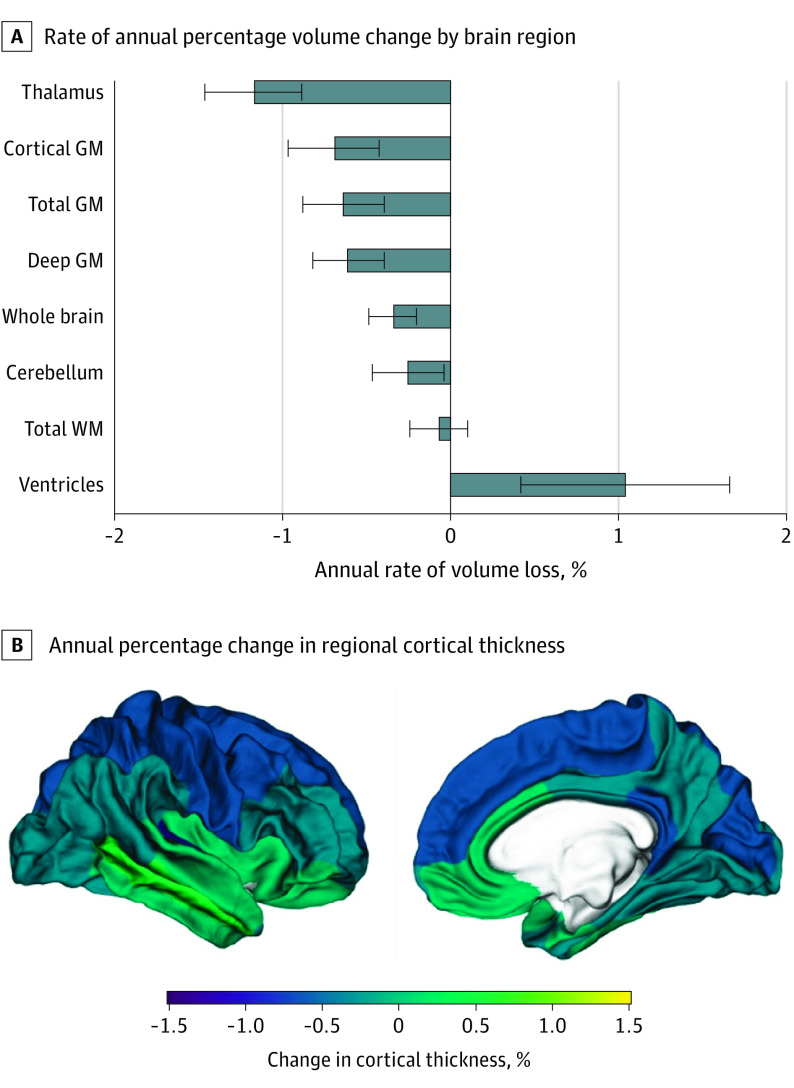
Annual Percentage Change in Volume and Regional Cortical Thickness GM indicates gray matter; TBV, total brain volume; WM, white matter.

All brain parenchymal volumes, except total WM, showed a significant rate of tissue loss during follow-up. The annual rate of TBV loss was −0.35% (95% CI, −0.49 to −0.20), and the highest regional rate was detected in the thalamus (−1.17%; 95% CI, −1.47 to −0.89). A significant longitudinal thinning was evident for whole CTh (−0.30%; 95% CI, −0.53 to −0.07), as well as for parietal, occipital, frontal, and insular cortical areas (Figure 2).

### Association Between MRI Measures at Baseline and Rates of Atrophy

Higher brain parenchymal fraction at baseline was associated with subsequent lower rates of loss in TBV (β, 0.087; 95% CI, 0.042 to 0.132; *P* = .009), total GM volume (β, 0.131; 95% CI, 0.042 to 0.220; *P* = .01), and cerebellar volume (β, 0.176; 95% CI, 0.110 to 0.244; *P* < .001) and with a lower rate of ventricular enlargement (β, −0.63; 95% CI, −0.100 to −0.027; *P* = .005) (eTable 3 in the Supplement).

Increased T2 lesion load at baseline was associated with subsequent higher rates of tissue loss of TBV (β, −0.098; 95% CI, −0.167 to −0.030; *P* = .03), total WM volume (β, −0.110; 95% CI, −0.168 to −0.053; *P* = .002), and thalamic volume (β, −0.130; 95% CI, −0.225 to −0.033; *P* = .03) and with accelerated ventricular enlargement (β, 0.171; 95% CI, 0.106 to 0.237; *P* < .001) (eTable 3 in the Supplement).

### Association Between MRI Lesion Activity and Rates of Atrophy

The rate of change in T2LV during follow-up was associated with the rates of loss of TBV (*b*, −0.13; 95% CI, −0.18 to −0.08; *P* < .001), total GM (*b*, −0.13; 95% CI, −0.22 to −0.05; *P* = .009), cortical GM (*b*, −0.14; 95% CI, −0.24 to −0.05; *P* = .009), deep GM (*b*, −0.11; 95% CI, −0.18 to −0.03; *P* = .01), total WM (*b*, −0.20; 95% CI, −0.25 to −0.14; *P* < .001), and thalamus (*b*, −0.17; 95% CI, −0.27 to −0.07; *P* = .003). Moreover, the accumulation of T2LV was associated with accelerated ventricular enlargement (*b*, 0.40; 95% CI, 0.20 to 0.61; *P* < .001). Similar results were obtained considering the annualized number of new and enlarged WM lesions (eTable 4 in the Supplement).

### Association Between Clinical Activity and Rates of Atrophy

The annualized relapse rate was associated with the rate of deep GM atrophy (*b*, −0.59; 95% CI, −0.97 to −0.20; *P* = .047). The rate of EDSS change during observation was associated with accelerated atrophy in the thalamus (*b*, −0.86; 95% CI, −1.35 to −0.37; *P* = .01) and faster ventricular enlargement (*b*, 1.69; 95% CI, 0.62 to 2.76; *P* = .01) (eTable 4 in the Supplement).

### Comparison Between Patients With PIRA and Stable Patients

During observation, 46 patients presented only PIRA activity with a total of 49 events of PIRA resulting in a median (IQR) annualized increase in EDSS score of 0.20 (0.11-0.50) points; this population was propensity score–matched with 46 stable patients (eTable 5 in the Supplement). The matched groups did not differ at baseline in disability and T2LV. Baseline brain parenchymal fraction was lower in patients with PIRA than in stable patients (median [IQR] in PIRA, 0.756 [0.715-0.788]; median [IQR] in stable, 0.769 [0.747-0.807]; *P* = .045). There was no difference in T2LV change during follow-up between groups.

**Figure 3.**
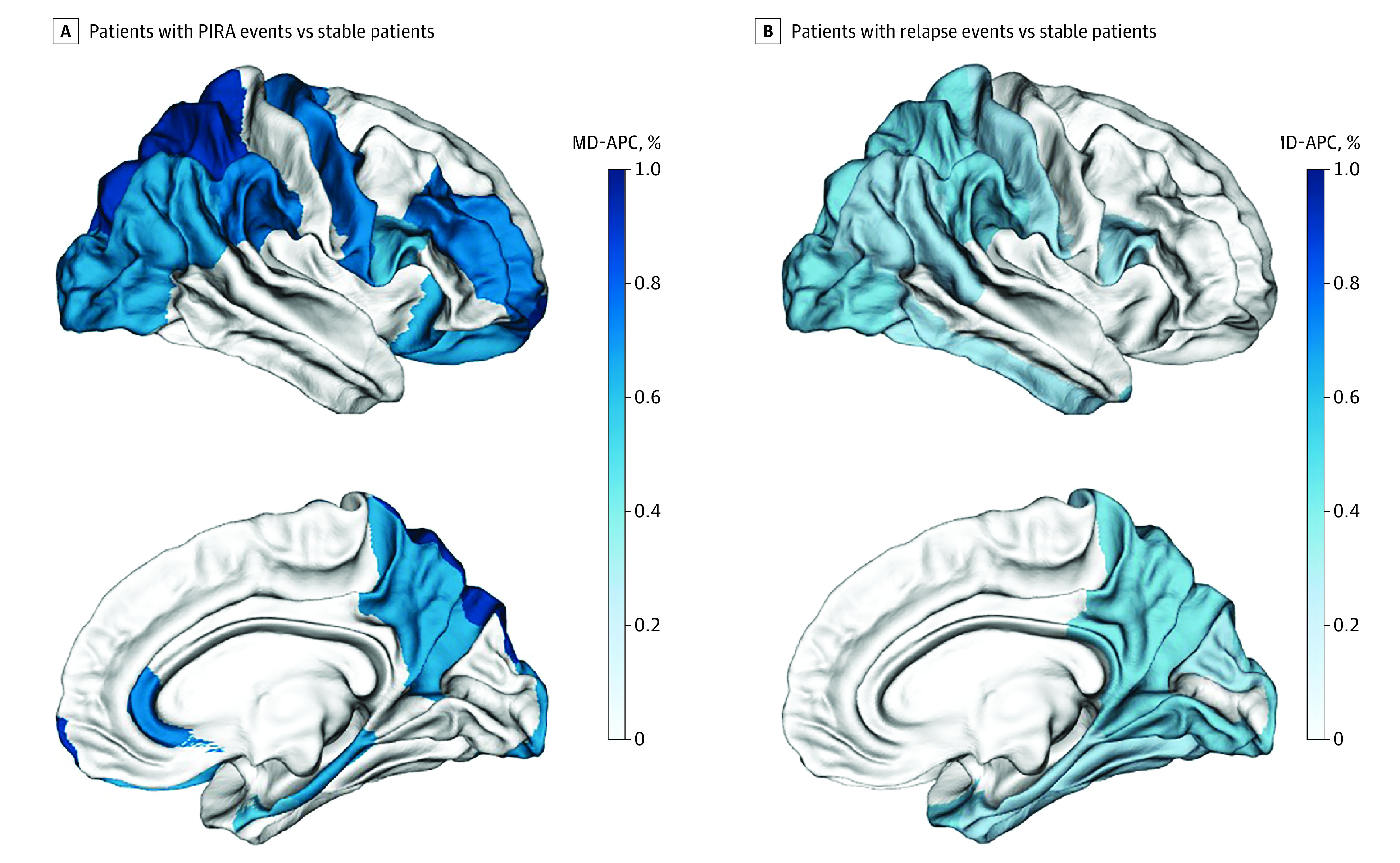
Differences in Rates of Regional Cortical Thinning Comparing Stable Patients vs Patients With Only Progression Independent of Relapse Activity (PIRA) or Patients With Only Relapse Activity The effect size, expressed as mean difference in annual percentage cortical thickness change (MD-APC), is graphically displayed in different shades of blue for each of the Desikan-Killiany atlas^25^ regions presenting significant differences between groups after correction for multiple comparisons.

Compared with stable patients, accelerated volume loss was detected in patients with PIRA for TBV (MD-APC, −0.36; 95% CI, −0.60 to −0.12; *P* = .02), total GM (MD-APC, −0.59; 95% CI, −1.00 to −0.18; *P* = .02), and cortical GM (MD-APC, −0.71; 95% CI, −1.18 to −0.24; *P* = .02); faster ventricular enlargement was also observed (MD-APC, 1.50; 95% CI, 0.47 to 2.55; *P* = .02). In addition, accelerated thinning was detected in the whole cortex (MD-APC, −0.62; 95% CI, −1.06 to −0.16; *P* = .02), as well as in each of the following cortical areas: temporal, frontal, parietal, insular, and cingulate (Table 2, Figure 3, and eFigures 1 and 2 in the Supplement).

**Table 2.** Differences in Brain Atrophy Rates for the Propensity Score–Matched Group Comparisons^a^

Brain structure	PIRA (n = 46) vs stable (n = 46)			PIRA without radiological inflammatory activity (n = 26) vs stable (n = 26)			Relapsing (n = 122) vs stable (n = 122)			PIRA (n = 46) vs relapsing (n = 46)	
	MD-APC (95% CI)	*P* value	FDR *P*	MD-APC (95% CI)	*P* value	FDR *P*	MD-APC (95% CI)	*P* value	FDR *P*	MD-APC (95% CI)	*P* value^b^
Total brain volume	−0.359 (−0.596 to −0.119)	.004	.02	−0.485 (−0.725 to −0.231)	.01	.03	−0.182 (−0.343 to −0.020)	.03	.04	−0.144 (−0.426 to 0.139)	.32
Total GM volume	−0.590 (−1.001 to −0.182)	.005	.02	−0.831 (−1.344 to −0.267)	.002	.02	−0.324 (−0.587 to −0.058)	.02	.04	−0.034 (−0.474 to 0.406)	.88
Total WM volume	0.064 (−0.318 to 0.460)	.74	.74	−0.089 (−0.644 to 0.437)	.56	.56	−0.048 (−0.242 to 0.147)	.63	.67	−0.153 (−0.508 to 0.206)	.40
Cortical GM volume	−0.712 (−1.184 to −0.242)	.004	.02	−0.998 (−1.464 to −0.398)	.004	.02	−0.330 (−0.614 to −0.044)	.02	.04	−0.077 (−0.574 to 0.421)	.76
Deep GM volume	−0.196 (−0.519 to 0.127)	.24	.28	−0.346 (−0.766 to 0.082)	.12	.15	−0.314 (−0.571 to −0.055)	.02	.04	0.073 (−0.309 to 0.455)	.71
Thalamic volume	−0.237 (−0.738 to 0.264)	.36	.39	−0.406 (−1.055 to 0.252)	.34	.36	−0.335 (−0.670 to 0.011)	.06	.08	0.089 (−0.485 to 0.666)	.76
Ventricular system volume	1.502 (0.473 to 2.553)	.006	.02	1.816 (0.506 to 3.149)	.01	.03	0.322 (−0.412 to 1.061)	.39	.45	−0.666 (−0.016 to 2.465)	.06
Cerebellar volume	−0.258 (−0.602 to 0.077)	.14	.18	−0.317 (−0.744 to 0.106)	.16	.18	0.015 (−0.212 to 0.242)	.90	.90	−0.143 (−0.512 to 0.227)	.45
Mean CTh	−0.617 (−1.058 to −0.164)	.01	.02	−0.828 (−1.362 to −0.267)	.006	.02	−0.306 (−0.570 to −0.041)	.03	.04	−0.044 (−0.478 to 0.390)	.84
Temporal CTh	−0.487 (−0.861 to −0.078)	.02	.03	−0.591 (−1.045 to −0.108)	.03	.046	−0.342 (−0.577 to −0.108)	.005	.03	−0.085 (−0.341 to 0.510)	.69
Frontal CTh	−0.673 (−1.181 to −0.155)	.01	.02	−1.041 (−1.573 to −0.484)	.001	.01	−0.234 (−0.542 to 0.077)	.14	.18	−0.127 (−0.614 to 0.367)	.61
Parietal CTh	−0.713 (−1.210 to −0.216)	.007	.02	−0.829 (−1.433 to −0.195)	.02	.03	−0.404 (−0.682 to −0.125)	.005	.03	−0.061 (−0.540 to 0.416)	.80
Occipital CTh	−0.544 (−1.112 to 0.005)	.06	.08	−0.739 (−1.621 to 0.104)	.10	.14	−0.441 (−0.738 to −0.146)	.004	.03	0.367 (−0.135 to 0.870)	.15
Insular CTh	−0.617 (−1.058 to −0.164)	.01	.02	−0.528 (−1.018 to 0.016)	.04	.06	−0.306 (−0.570 to 0.041)	.03	.04	−0.044 (−0.478 to 0.390)	.84
Cingulate CTh	−0.573 (−1.036 to −0.107)	.02	.03	−0.789 (−1.355 to −0.198)	.01	.03	−0.280 (−0.521 to −0.040)	.02	.04	−0.075 (−0.523 to 0.372)	.74

Abbreviations: CTh, cortical thickness; FDR, false discovery rate; GM, gray matter; MD-APC, mean difference in annual percentage change; PIRA, progression independent of relapse activity; WM, white matter.

^a^ Associations between rates of atrophy (dependent variables) and clinical group were investigated as the interaction term between clinical group and time in linear mixed-effect models, including total intracranial volume as covariate, random intercepts (for participants and magnetic resonance imaging protocols), and a random slope (on time).

^b^ The FDR *P* value for every structure was *P* = .88.

Accelerated atrophy rates were also observed in the subgroup of patients with PIRA who did not have radiological inflammatory activity during the entire follow-up (n = 26) when compared with a matched group of stable patients.

### Comparison Between Patients With Relapse Activity and Stable Patients

During follow-up, 122 patients had relapse activity without PIRA events; this population was propensity score–matched with 122 stable patients (eTable 6 in the Supplement). There were no differences between groups at baseline in disability, T2LV, and brain volumetric measurements. Mean (SD) annualized relapse rate in the group with relapses was 0.48 (0.43).

Compared with clinically stable patients, patients with only relapse activity showed increased atrophy rates in TBV (MD-APC, −0.18; 95% CI, −0.34 to −0.02; *P* = .04) and total GM (MD-APC, −0.32; 95% CI, −0.59 to −0.06; *P* = .04), which were evident both in cortical GM (MD-APC, −0.33; 95% CI, −0.61 to −0.04; *P* = .04) and deep GM (MD-APC, −0.31; 95% CI, −0.57 to −0.06; *P* = .04). Accelerated thinning was detected in the whole cerebral cortex (MD-APC, −0.31; 95% CI, −0.57 to −0.04; *P* = .04), as well as in the cortex of the temporal, parietal, occipital, insular, and cingulate lobes (Table 2 and Figure 3). Among the 122 patients with only relapse activity, 35 had confirmed disability progression during follow-up. Atrophy rates did not differ between patients with and without confirmed disability progression in any of the regions investigated.

### Comparison Between Patients With PIRA and Patients With Relapse Activity

After propensity score matching (eTable 7 and eTable 8 in the Supplement), no significant differences in atrophy rates were detected between patients experiencing only PIRA events and patients who had only relapse activity (Table 2).

## Discussion

In this large longitudinal cohort study, we show that patients with RMS and PIRA exhibit increased rates of tissue loss in several brain areas compared with patients who are clinically stable. Our data also indicate that patients with RMS and PIRA are subject to global brain tissue loss similar to that of patients experiencing relapse activity.

To selectively investigate PIRA, we identified patients with confirmed disability progression who were free from relapses during the entire observation period. When compared with a propensity score–matched population of clinically stable patients, patients with PIRA showed a remarkable increase in TBV atrophy, mainly driven by GM loss. Accelerated tissue loss was evident in cortical volume and cortical thickness, especially in frontal and parietal areas. Notably, the 2 groups did not differ in longitudinal radiological inflammatory activity. Moreover, the results were confirmed in a subgroup of patients with PIRA who were completely free from radiological inflammatory activity for the entire follow-up. In clinical practice, the occurrence of PIRA in patients with RMS often remains unrecognized because patients with low levels of disability are infrequently considered to potentially present a progressive disease course.^4^

Our results showing an association between PIRA and diffuse neurodegeneration provide strong evidence of the need to promptly recognize PIRA, to prevent the accrual of irreversible central nervous system tissue damage. Approved DMTs may differ in the ability to prevent disability accumulation due to PIRA,^4^ and escalation/induction strategies have not yet been assessed in patients with PIRA. Future clinical trials should therefore aim at identifying the best therapeutic strategy for these patients, both those with and those without relapses.

By reflecting ongoing tissue damage and destruction, brain volumetry has emerged as a useful measure to understand disease progression in MS: in fact, neuroaxonal loss exceeding the reserve capacity of the central nervous system is thought to be the ultimate driver of disability accumulation.^37^ Because of this, assessing brain volume loss is now recommended in trials evaluating drugs with potential neuroprotective effect, even as a primary outcome measure in progressive MS.^39^ Nonetheless, various technical and biological factors limit the measurement of longitudinal brain volume changes in the clinic.^39^ On the other hand, the association between baseline brain atrophy and the risk of PIRA suggests that cross-sectional measures of brain volume may help identify patients at risk of neurodegeneration.^6^

Several studies have previously failed in detecting significant longitudinal differences in atrophy rates between patients with RMS and those with progressive MS.^41^ Eshaghi et al^36^ reported increased atrophy but limited to temporal cortical GM in SPMS with respect to RMS. It might be speculated that the lack of striking differences between RMS and SPMS results from the uncertainty in the clinical definition of the disease course. Indeed, the traditional distinction between an initial relapse only and a secondary progressive course has been called into question by the evidence of PIRA in RMS.^4^ Concordantly, our results showed that patients with RMS may have excess neurodegeneration. Moreover, our data showing that patients with RMS and PIRA exhibit more pronounced brain atrophy than stable patients with RMS support the association of brain volume loss and disability progression in MS. Further, the fact that patients with PIRA show significantly increased brain atrophy compared with stable patients with RMS provides additional important evidence that neurodegenerative changes are more pronounced in a subgroup of patients with RMS than in others. Whether the presence of PIRA represents an early phase of SPMS or a milder form of progression should be investigated in future longitudinal studies.

As described previously,^44^ we found an association also between focal inflammatory activity and diffuse and regional atrophic changes. Lesions in MS may result in brain volume loss through (1) direct inflammatory damage leading to loss of myelin, oligodendrocytes, and axons within lesions and through (2) indirect tissue loss after Wallerian degeneration.^38^ Besides, in our cohort, higher T2LV at baseline was related to lower brain volumes and cortical thinning. Longitudinally, MRI lesion activity was linked to a diffuse increase in rates of tissue loss, involving both WM and GM, with the strongest association in the thalamus.

The association between acute inflammatory activity and atrophy was further corroborated by the evidence of accelerated tissue loss in patients exhibiting only relapse activity. Indeed, this population showed increased TBV loss with respect to a propensity score–matched clinically quiescent population. Interestingly, the acceleration in tissue loss was not seen with WM volume and was driven by both deep GM and cortical GM atrophy. As previously reported,^6^ we also found that patients with relapse activity with and without associated confirmed disability progression did not differ in atrophy rates.

Remarkably, no significant differences were detected when atrophy rates were compared between patients experiencing PIRA only and patients experiencing relapse activity only. Our findings are in line with and expand the results of Cree et al,^6^ who reported increased TBV loss in association to both silent disease progression and overt inflammatory activity, without significant differences between the two. Our study also showed that accelerated brain atrophy in patients with PIRA and in patients with relapse activity is mainly driven by GM atrophy, with involvement of the cerebral cortex in both patient groups and involvement of deep GM in relapsing patients only.

### Strengths and Limitations

Strengths of our investigation include the large sample size and the availability of prospectively performed standardized neurological assessments. In addition, brain volumetric analysis was performed with a pipeline optimized for longitudinal analyses,^24^ requiring long computational processes and manual editing but providing sophisticated evaluations and representing the gold standard in CTh measurements.^47^

Our study also presents some limitations. First, the inclusion of MRI data acquired with different protocols may have represented a confounding factor. However, as part of the Swiss Multiple Sclerosis Cohort study, optimization was performed of scans’ signal-to-noise ratio among different centers. Moreover, statistical analysis accounted for heterogeneity in MRI protocols as a confounding factor.

In our study, we did not include measurements of spinal cord atrophy and cortical lesions. Both may help further characterize the mechanisms underlying PIRA, as they have been previously shown to predict physical disability and disease progression,^48^ silent progression, and conversion to SPMS.^50^ In addition, the criterion used to determine PIRA was based only on EDSS score. Given the absence of measures of upper and lower extremity function in our cohort, subtle neurological worsening that did not result in EDSS score increase may have been overlooked. Furthermore, DMTs may have constituted a bias in our study, which we tried to overcome by performing propensity score matching for treatment status and considering treatment groups in sensitivity analyses.

## Conclusions

Our data show that events of insidious PIRA are associated with increased atrophy rates, likely reflecting ongoing diffuse neurodegenerative processes, especially in cortical GM. These results point to the need to promptly identify patients with PIRA in clinical practice, because they may benefit from optimized therapeutic regimens. In this context, clinical trials to assess the potential benefit of treatment escalation/induction in patients with RMS and PIRA are warranted.

## Notes

Supplement. **eTable 1.** Overview of MRI protocols **eTable 2.** Association between disability (EDSS), and T2-lesion load (Log-T2LV), with brain measurements at baseline **eTable 3.** Association between baseline brain parenchymal fraction, and baseline T2-lesion load, with atrophy rates **eTable 4.** Association between rates of atrophy and MRI and clinical activity **eTable 5.** Groups’ characteristics before and after propensity-score matching: patients with progression independent of relapse activity and without relapses (PIRA) vs patients with clinical stability (Stable) **eTable 6. **Groups’ characteristics before and after propensity-score matching: patients with relapse activity and without PIRA (Relapsing) vs patients with clinical stability (Stable) **eTable 7. **Groups’ characteristics before and after propensity-score matching: patients with progression independent of relapse activity and without relapses (PIRA) vs patients with relapse activity and without PIRA (Relapsing) **eTable 8.** Atrophy rates in clinical subgroups **eFigure 1. **Annualized total brain volume loss in clinical subgroups **eFigure 2. **Annualized total gray matter volume loss in clinical subgroups
eMethods

eReferences
