# Supplementary material for: Association of Brain Atrophy With Disease Progression Independent of Relapse Activity in Patients With Relapsing Multiple Sclerosis
Source: JAMA Neurol. 2022 May 16:e221025. Online ahead of print. doi: 10.1001/jamaneurol.2022.1025 (PMC9112138; doi:10.1001/jamaneurol.2022.1025)
Supplement: Supplement. — eTable 1. Overview of MRI protocols eTable 2. Association between disability (EDSS), and T2-lesion load (Log-T2LV), with brain measurements at baseline eTable 3. Association between baseline brain parenchymal fraction, and baseline T2-lesion load, with atrophy rates eTable 4. Association between rates of atrophy and MRI and clinical activity eTable 5. Groups’ characteristics before and after propensity-score matching: patients with progression independent of relapse activity and without relapses (PIRA) vs patients with clinical stability (Stable) eTable 6. Groups’ characteristics before and after propensity-score matching: patients with relapse activity and without PIRA (Relapsing) vs patients with clinical stability (Stable) eTable 7. Groups’ characteristics before and after propensity-score matching: patients with progression independent of relapse activity and without relapses (PIRA) vs patients with relapse activity and without PIRA (Relapsing) eTable 8. Atrophy rates in clinical subgroups eFigure 1. Annualized total brain volume loss in clinical subgroups eFigure 2. Annualized total gray matter volume loss in clinical subgroups eMethods eReferences [file jamaneurol-e221025-s001.pdf]

Supplemental Online Content

Cagol A, Schaedelin S, Barakovic M, et al. Association of brain atrophy with disease progression independent of relapse activity in patients with relapsing multiple sclerosis. *JAMA Neurol*. Published online May 16, 2022. doi:10.1001/jamaneurol.2022.1025

eTable 1. Overview of MRI protocols____page 2

eTable 2. Association between disability (EDSS), and T2-lesion load (Log-T2LV), with brain measurements at baseline____page 3

eTable 3. Association between baseline brain parenchymal fraction, and baseline T2-lesion load, with atrophy rates____page 4

eTable 4. Association between rates of atrophy and MRI and clinical activity____page 5

eTable 5. Groups' characteristics before and after propensity-score matching: patients with progression independent of relapse activity and without relapses (PIRA) vs patients with clinical stability (Stable)____page 7

eTable 6. Groups' characteristics before and after propensity-score matching: patients with relapse activity and without PIRA (Relapsing) vs patients with clinical stability (Stable)____page 8

eTable 7. Groups' characteristics before and after propensity-score matching: patients with progression independent of relapse activity and without relapses (PIRA) vs patients with relapse activity and without PIRA (Relapsing)____page 9

eTable 8. Atrophy rates in clinical subgroups____page 10

eFigure 1. Annualized total brain volume loss in clinical subgroups____page 11

eFigure 2. Annualized total gray matter volume loss in clinical subgroups____page 11

eMethods____page 12

Reliability in brain volumetric measures obtained with different software packages
Sensitivity analysis: effect of disease-modifying therapies

eReferences____page 13

This supplemental material has been provided by the authors to give readers additional information about their work.

© 2022 Cagol A et al. *JAMA Neurol*.

eTable 1. Overview of MRI protocols

T1-weighted acquisition										
Center	Geneva	Bern		Basel		Aarau		Lugano	St. Gallen	
Nr of scans (subjects)	188 (60)	31 (13)		1414 (359)		44 (19)		20 (9)	207 (56)	
Scanner vendor	Siemens	Siemens	Siemens	Siemens	Siemens	Siemens	Siemens	Siemens	Siemens	Siemens
Scanner model	Aera	Avanto	Avanto_fit	Avanto	Skyra_fit	Avanto	Avanto_fit	Skyra	Avanto	Avanto_fit
Magnetic field	1.5 Tesla	1.5 Tesla	1.5 Tesla	1.5 Tesla	3 Tesla	1.5 Tesla	1.5 Tesla	3 Tesla	1.5 Tesla	1.5 Tesla
TR (ms)	2200	1830	1720	2700	2300	2700	2700	1800	2700	2700
TE (ms)	2.67	2.92	2.92	5.03	3.02	5.03	5.03	2.43	3.08	3.09
Inversion time (ms)	900	1100	1100	950	900	950	950	900	950	950
Matrix size	256x256	256x256	256x256	256x256	250x240	256x256	256x256	256x256	256x256	256x256
FOV	256x256	256x256	256x256	256x256	250x240	256x256	256x256	256x256	256x256	256x256
Resolution (mm)	1x1x1	1x1x1	1x1x1	1x1x1	1x1x1	1x1x1	1x1x1	1x1x1	1x1x1	1x1x1
Flip angle	8	15	15	8	9	8	8	9	8	8
Acquisition type	3D	3D	3D	3D	3D	3D	3D	3D	3D	3D

Abbreviations: TR, repetition time; TE, echo time; TI, inversion time; FOV, field of view; FLAIR, fluid attenuated inversion recovery.

© 2022 Cagol A et al. *JAMA Neurol*.

eTable 2. Association between disability (EDSS), and T2-lesion load (Log-T2LV), with brain measurements at baseline.

	EDSS			Log-T2LV		
Brain structure	*β (95% CI)*	*p-value*	*p-adjusted (FDR)*	*β (95% CI)*	*p-value*	*p-adjusted (FDR)*
Total brain volume	-0.081 (-0.134; -0.028)	0.0028	**0.0140**	-0.136 (-0.186; -0.087)	<0.0001	**<0.0001**
Total GM volume	-0.074 (-0.133; -0.017)	0.0129	**0.0386**	-0.094 (-0.149; -0.039)	0.0009	**0.0012**
Total WM volume	-0.069 (-0.125; -0.012)	0.0180	**0.0412**	-0.154 (-0.207; -0.101)	<0.0001	**<0.0001**
Cortical GM volume	-0.052 (-0.144; 0.009)	0.0998	0.1871	-0.079 (-0.138; 0.021)	0.0080	**0.0092**
Deep GM volume	-0.174 (-0.240; -0.105)	<0.0001	**<0.0001**	-0.318 (-0.377; -0.258)	<0.0001	**<0.0001**
Thalamic volume	-0.187 (-0.255; -0.120)	<0.0001	**<0.0001**	-0.354 (-0.412; -0.295)	<0.0001	**<0.0001**
Ventricular system volume	0.110 (0.033; 0.188)	0.0059	**0.0220**	0.259 (0.187; 0.330)	<0.0001	**<0.0001**
Cerebellar volume	-0.095 (-0.175; -0.016)	0.0192	**0.0412**	-0.001 (-0.077; 0.074)	0.9750	0.9750
Mean CTh	-0.032 (-0.112; 0.045)	0.4170	0.5331	-0.162 (-0.235; -0.090)	<0.0001	**<0.0001**
Temporal CTh	-0.033 (-0.118; 0.049)	0.4320	0.5331	-0.159 (-0.237; -0.082)	<0.0001	**0.0001**
Frontal CTh	-0.029 (-0.109; 0.048)	0.4620	0.5331	-0.109 (-0.182; -0.036)	0.0039	**0.0049**
Parietal CTh	-0.064 (-0.147; 0.017)	0.1250	0.2083	-0.174 (-0.250; -0.098)	<0.0001	**<0.0001**
Occipital CTh	-0.012 (-0.088; 0.064)	0.7603	0.7603	-0.177 (-0.247; -0.107)	<0.0001	**<0.0001**
Insular CTh	-0.032 (-0.112; 0.045)	0.4170	0.5331	-0.162 (-0.235; -0.090)	<0.0001	**<0.0001**
Cingulate CTh	0.021 (-0.061; 0.103)	0.6100	0.6536	-0.102 (-0.180; -0.024)	0.0108	**0.0116**

Associations between brain measurements at baseline (dependent variable) and the independent variables of interest were assessed in linear mixed effect models adjusting for total intracranial volume (TIV), sex, age, and disease duration – as fixed-effect covariates – and MRI protocol – as random intercept.

Abbreviations: EDSS, Expanded Disability Status Scale; Log-T2LV, logarithmic transformation of T2-lesion volume; β, standardized beta coefficient; CI, confidence interval; FDR, false discovery rate; GM, gray matter; WM, white matter; CTh, cortical thickness.

© 2022 Cagol A et al. *JAMA Neurol*.

eTable 3. Association between baseline brain parenchymal fraction, and baseline T2-lesion load, with atrophy rates.

	Baseline BPF			Baseline Log-T2LV		
Brain structure	*β (95% CI)*	*p-value*	*p-adjusted (FDR)*	*β (95% CI)*	*p-value*	*p-adjusted (FDR)*
Total brain volume	0.087 (0.042; 0.132)	0.0018	**0.0091**	-0.098 (-0.167; -0.030)	0.0053	**0.0266**
Total GM volume	0.131 (0.042; 0.220)	0.0039	**0.0147**	-0.076 (-0.212; 0.061)	0.2797	0.5245
Total WM volume	0.047 (0.008; 0.087)	0.0196	0.0588	-0.110 (-0.168; -0.053)	0.0002	**0.0017**
Cortical GM volume	0.095 (0.003; 0.189)	0.0442	0.0947	-0.038 (-0.178; 0.104)	0.6009	0.8550
Deep GM volume	0.056 (0.003; 0.110)	0.0378	0.0944	-0.100 (-0.193; -0.008)	0.0353	0.1058
Thalamic volume	0.030 (-0.026; 0.086)	0.2983	0.3888	-0.130 (-0.225; -0.033)	0.0080	**0.0302**
Ventricular system volume	-0.063 (-0.100; -0.027)	0.0007	**0.0053**	0.171 (0.106; 0.237)	<0.0001	**<0.0001**
Cerebellar volume	0.176 (0.110; 0.244)	<0.0001	**<0.0001**	-0.096 (-0.193; 0.001)	0.0543	0.1358
Mean CTh	0.075 (-0.078; 0.229)	0.3370	0.3888	0.032 (-0.176; 0.239)	0.7630	0.8550
Temporal CTh	0.045 (-0.100; 0.190)	0.5430	0.5818	-0.025 (-0.217; 0.166)	0.7980	0.8550
Frontal CTh	0.079 (-0.074; 0.233)	0.3128	0.3888	0.130 (-0.078; 0.340)	0.2222	0.4761
Parietal CTh	0.096 (-0.55; 0.248)	0.2158	0.3596	-0.010 (-0.217; 0.196)	0.9216	0.9216
Occipital CTh	-0.080 (-0.217; 0.056)	0.2459	0.3688	-0.074 (-0.258; 0.109)	0.4311	0.7185
Insular CTh	0.095 (-0.078; 0.229)	0.1830	0.3431	0.032 (-0.176; 0.239)	0.7630	0.8550
Cingulate CTh	0.029 (-0.092; 0.151)	0.6370	0.6370	-0.033 (-0.205; 0.139)	0.7092	0.8550

The associations were investigated as the interaction term between the independent variables of interest (baseline BPF and baseline log-T2LV) and time in linear mixed effect models including as covariates total intracranial volume (TIV), sex, age at baseline, disease duration at baseline, and the interactions between sex and baseline disease duration with time. Models included both random intercepts (for subjects and MRI protocols), and a random slope (on time).
Abbreviations: BPF, brain parenchymal fraction; Log-T2LV, logarithmic transformation of T2-lesion volume; β, standardized beta coefficient; CI, confidence interval; FDR, false discovery rate; GM, gray matter; WM, white matter; CTh, cortical thickness."

© 2022 Cagol A et al. *JAMA Neurol*.

eTable 4. Association between rates of atrophy and MRI and clinical activity.

	**Rate of T2LV change**			**Rate of new/enlarged WMLs**			**ARR**			**Rate of EDSS change**		
Brain structure	*Estimate (95% CI)*	*p-value*	*p-adjusted (FDR)*	*Estimate (95% CI)*	*p-value*	*p-adjusted (FDR)*	*Estimate (95% CI)*	*p-value*	*p-adjusted (FDR)*	*Estimate (95% CI)*	*p-value*	*p-adjusted (FDR)*
Total brain volume	-0.131 (-0.179; -0.082)	<0.0001	**<0.0001**	-0.047 (-0.067; -0.027)	<0.0001	**<0.0001**	-0.310 (-0.571; -0.047)	0.0213	0.0699	-0.274 (-0.539; 0.012)	0.0415	0.1558
Total GM volume	-0.129 (-0.216; -0.045)	0.0030	**0.0088**	-0.039 (-0.074; -0.004)	0.0324	0.0607	-0.466 (-0.911; -0.016)	0.0425	0.0890	-0.376 (-0.826; 0.070)	0.1015	0.2538
Total WM volume	-0.197 (-0.254; -0.139)	<0.0001	**<0.0001**	-0.068 (-0.092; -0.044)	<0.0001	**<0.0001**	-0.204 (-0.525; 0.118)	0.2150	0.2481	-0.236 (-0.557; 0.083)	0.1470	0.2923
Cortical GM volume	-0.142 (-0.237; -0.046)	0.0041	**0.0088**	-0.032 (-0.072; 0.008)	0.1149	0.1915	-0.483 (-0.979; 0.018)	0.0593	0.0890	-0.347 (-0.850; 0.151)	0.1754	0.2923
Deep GM volume	-0.106 (-0.177; -0.033)	0.0035	**0.0099**	-0.078 (-0.107; -0.048)	<0.0001	**<0.0001**	-0.588 (-0.974; -0.201)	0.0031	**0.0465**	-0.348 (-0.712; 0.018)	0.0629	0.1887
Thalamic volume	-0.166 (-0.265; -0.070)	0.0007	**0.0028**	-0.096 (-0.137; -0.055)	<0.0001	**<0.0001**	-0.651 (-1.172; -0.110)	0.0169	0.0699	-0.859 (-1.350; -0.371)	0.0007	**0.0099**
Ventricular system volume	0.401 (0.195; 0.606)	0.0001	**0.0006**	0.198 (0.111; 0.286)	<0.0001	**<0.0001**	0.618 (-0.534; 1.788)	0.2969	0.2969	1.686 (0.622; 2.756)	0.0019	**0.0141**
Cerebellar volume	-0.010 (-0.084; 0.065)	0.8053	0.8053	-0.015 (-0.045; 0.015)	0.3402	0.3925	0.222 (-0.170; 0.614)	0.2700	0.2893	-0.496 (-0.886; 0.106)	0.0135	0.0675
Mean CTh	-0.059 (-0.142; 0.024)	0.1649	0.2195	-0.024 (-0.057; 0.010)	0.1670	0.2277	-0.417 (-0.841; 0.010)	0.0564	0.0890	-0.177 (-0.614; 0.259)	0.4282	0.4996

© 2022 Cagol A et al. *JAMA Neurol*.

	Rate of T2LV change			Rate of new/enlarged WMLs			ARR			Rate of EDSS change		
Temporal CTh	-0.082 (-0.156; -0.009)	0.0281	0.0527	-0.037 (-0.066; -0.007)	0.0155	**0.0388**	-0.383 (-0.756 0.006)	0.0473	0.0890	-0.155 (-0.543; 0.232)	0.4330	0.4996
Frontal CTh	-0.067 (-0.165; 0.029)	0.1756	0.2195	-0.025 (-0.064; 0.015)	0.2199	0.2749	-0.335 (-0.834; 0.169)	0.1927	0.2409	-0.088 (-0.600; 0.422)	0.7367	0.7367
Parietal CTh	-0.042 (-0.133; 0.047)	0.3530	0.4073	-0.014 (-0.050; 0.022)	0.4408	0.4723	-0.531 (-0.984; 0.074)	0.0233	0.0699	-0.255 (-0.724; 0.212)	0.2863	0.4295
Occipital CTh	0.039 (-0.059; 0.138)	0.4315	0.4624	0.009 (-0.031; 0.048)	0.6619	0.6619	-0.687 (-1.182; 0.190)	0.0073	0.0548	-0.103 (-0.620; 0.417)	0.6956	0.7367
Insular CTh	-0.059 (-0.142; 0.024)	0.1649	0.2195	-0.024 (-0.057; 0.010)	0.1670	0.2277	-0.417 (-0.841; 0.010)	0.0564	0.0890	-0.177 (-0.614; 0.259)	0.4282	0.4996
Cingulate CTh	-0.088 (-0.169; 0.008)	0.0318	0.0530	-0.039 (-0.072; -0.007)	0.0193	**0.0414**	-0.391 (-0.809; 0.029)	0.0699	0.0953	-0.299 (-0.720; 0.124)	0.1672	0.2923

Associations between rates of atrophy (dependent variables) and the independent variables of interests were investigated as the interaction term between the independent variables and time in linear mixed effect models including as covariates total intracranial volume (TIV), sex, age at baseline, disease duration at baseline, and the interactions between sex and baseline disease duration with time. Models included both random intercepts (for subjects and MRI protocols), and a random slope (on time).
Abbreviations: T2LV, T2-lesion volume; WMLs, white matter lesions; ARR, annualized relapse rate; EDSS, Expanded Disability Status Scale; FDR, false discovery rate; GM, gray matter; WM, white matter; CTh, cortical thickness.

© 2022 Cagol A et al. *JAMA Neurol*.

eTable 5 Groups' characteristics before and after propensity-score matching: patients with progression independent of relapse activity and without relapses (PIRA) vs patients with clinical stability (Stable).

	Before matching			After matching		
	PIRA	Stable	Comparison	PIRA	Stable	Comparison
Follow-up duration: median (IQR)	4.00 (2.02-4.96)	2.97 (1.54-4.25)	Z=-2.014; p=0.0444	4.00 (2.02-4.96)	4.07 (2.49-5.14)	Z=-0.918; p=0.3576
Age: median (IQR)	45.6 (36.5-53.7)	41.5 (33.0-49.8)	Z=-2.145; p=0.0324	45.6 (36.5-53.7)	45.6 (36.1-50.3)	Z=0.6325; p=0.5287
Female: %	73.9	62.6	X^2=2.2545; p=0.1332	73.9	78.3	X^2=0.239; p=0.6250
Disease duration: median (IQR)	10.0 (4.2-15.0)	7.1 (2.9-12.3)	Z=-1.531; p=0.1260	10.0 (4.2-15.0)	8.3 (3.2-15.5)	Z=0.3397; p=0.7279
Number of scans per patient: median (IQR)	4 (3-5)	3 (2-5)	Z=-2.718; p=0.0065	4 (3-5)	5 (3-5)	Z=-0.6091; p=0.5419
On DMTs: %	80.0	81.4	X^2=0.047; p=0.8280	80.0	87.0	X^2=0.7169; p=0.3972
Baseline BPF: median (IQR)	0.756 (0.715; 0.788)	0.775 (0.738; 0.807)	Z=2.362; p=0.0183	0.756 (0.715; 0.788)	0.769 (0.747; 0.807)	Z=-1.995; p=0.0455
Baseline T2LV: median (IQR)	5.7 (1.8; 15.1)	3.8 (1.6; 11.5)	Z=-0.863; p=0.3898	5.7 (1.8; 15.1)	3.6 (1.5; 11.0)	Z=0.902; p=0.3681
Annualized ΔT2LV, median (IQR), ml	-0.02 (-0.20; 0.73)	0.03 (-0.28; 0.37)	Z=-0.449; p=0.6527	-0.02 (-0.20; 0.73)	0.03 (-0.14; 0.25)	Z=0.215; p=0.8337
Sample size	46	334	/	46	46	/

Group comparisons were performed with Mann-Whitney U test and Chi-square test.
Abbreviations: PIRA, progression independent of relapse activity; IQR, interquartile range; DMTs, disease-modifying therapies; BPF, brain parenchymal fraction; T2LV, T2-lesion volume; ΔT2LV, change in T2-lesion volume.

© 2022 Cagol A et al. *JAMA Neurol*.

eTable 6. Groups' characteristics before and after propensity-score matching: patients with relapse activity and without PIRA (Relapsing) vs patients with clinical stability (Stable).

	**Before matching**			**After matching**		
	Relapsing	Stable	Comparison	Relapsing	Stable	Comparison
Follow-up duration: median (IQR)	3.97 (2.95-5.04)	2.97 (1.54-4.25)	Z=-4.671; p<0.0001	3.97 (2.95-5.04)	4.01 (2.96-5.04)	Z=-0.200; p=0.8415
Age: median (IQR)	38.3 (31.3-46.0)	41.5 (33.0-49.8)	Z=2.225; p=0.0257	38.3 (31.3-46.0)	38.1 (31.5-46.4)	Z=-0.251; p=0.8026
Female: %	77.0	62.6	X^2=8.397; p=0.0038	77.0	78.7	X^2=0.095; p=0.7578
Disease duration: median (IQR)	7.8 (3.0-13.2)	7.1 (2.9-12.3)	Z=-0.753; p=0.4533	7.8 (3.0-13.2)	6.7 (2.7-12.1)	Z=0.813; p=0.4179
Number of scans per patient: median (IQR)	4 (3-5)	3 (2-5)	Z=-3.408; p=0.0006	4 (3-5)	4 (3-5)	Z=-0.522; p=0.6031
On DMTs: %	84.4	81.4	X^2=0.547; p=0.4597	84.4	86.9	X^2=0.300; p=0.5838
Baseline BPF: median (IQR)	0.789 (0.764; 0.812)	0.775 (0.738; 0.807)	Z=-2.815; p=0.0048	0.789 (0.764; 0.812)	0.788 (0.756; 0.809)	Z=0.857; p=0.3898
Baseline T2LV: median (IQR)	4.5 (1.1; 12.1)	3.8 (1.6; 11.5)	Z=0.246; p=0.8026	4.5 (1.1; 12.1)	3.7 (1.7; 10.2)	Z=0.187; p=0.8493
Annualized ΔT2LV, median (IQR), ml	0.10 (-0.12; 0.85)	0.03 (-0.28; 0.37)	Z=-2.731; p=0.0063	0.10 (-0.12; 0.85)	0.03 (-0.26; 0.31)	Z=2.658; p=0.0078
Sample size	122	334	/	122	122	

Group comparisons were performed with Mann-Whitney U test and Chi-square test.
Abbreviations: PIRA, progression independent of relapse activity; IQR, interquartile range; DMTs, disease-modifying therapies; BPF, brain parenchymal fraction; T2LV, T2-lesion volume; ΔT2LV, change in T2-lesion volume.

© 2022 Cagol A et al. *JAMA Neurol*.

eTable 7. Groups' characteristics before and after propensity-score matching: patients with progression independent of relapse activity and without relapses (PIRA) vs patients with relapse activity and without PIRA (Relapsing).

	**Before matching**			**After matching**		
	PIRA	Relapsing	Comparison	PIRA	Relapsing	Comparison
Follow-up duration: median (IQR)	4.00 (2.02-4.96)	3.97 (2.95-5.04)	Z=0.752; p=0.4533	4.00 (2.02-4.96)	3.31 (2.62-4.88)	Z=0.129; p=0.8966
Age: median (IQR)	45.6 (36.5-53.7)	38.3 (31.3-46.0)	Z=-3.313; p=0.0009	45.6 (36.5-53.7)	45.8 (37.6-50.0)	Z=0.426; p=0.6672
Female: %	73.9	77.0	X^2=0.181; p=0.6704	73.9	73.9	X^2=0; p=1
Disease duration: median (IQR)	10.0 (4.2-15.0)	7.8 (3.0-13.2)	Z=-0.996; p=0.317	10.0 (4.2-15.0)	7.9 (3.1-12.4)	Z=0.7848; p=0.4354
Number of scans per patient: median (IQR)	4 (3-5)	4 (3-5)	Z=-0.553; p=0.5823	4 (3-5)	4 (3-5)	Z=-0.008; p=0.9920
On DMTs: %	80.0	84.4	X^2=0.383; p=0.5359	80.0	80.0	X^2=0; p=1
Baseline BPF: median (IQR)	0.756 (0.715; 0.788)	0.789 (0.764; 0.812)	Z=3.897; p=0.0001	0.756 (0.715; 0.788)	0.784 (0.763; 0.805)	Z=-2.690; p=0.0071
Baseline T2LV: median (IQR)	5.7 (1.8; 15.1)	4.5 (1.1; 12.1)	Z=-1.014; p=0.3125	5.7 (1.8; 15.1)	4.2 (1.3-12.6)	Z=0.707; p=0.4777
Annualized ΔT2LV, median (IQR), ml	-0.02 (-0.20; 0.73)	0.10 (-0.12; 0.85)	Z=1.154; p=0.2501	-0.02 (-0.20; 0.73)	0.03 (-0.17; 0.83)	Z=-0.316; p=0.749
Sample size	46	122	/	46	46	/

Group comparisons were performed with Mann-Whitney U test and Chi-square test.
Abbreviations: PIRA, progression independent of relapse activity; IQR, interquartile range; DMTs, disease-modifying therapies; BPF, brain parenchymal fraction; T2LV, T2-lesion volume; ΔT2LV, change in T2-lesion volume.

© 2022 Cagol A et al. *JAMA Neurol*.

eTable 8. Atrophy rates in clinical subgroups

	PIRA (n=46) vs Stable (n=46)			Relapsing (n=122) vs Stable (n=122)		
Brain structure	APC PIRA: mean (95% CI)	APC Stable: mean (95% CI)	*Comparison (p-value)*	APC Relapsing: mean (95% CI)	APC Stable: mean (95% CI)	*Comparison (p-value)*
Total brain volume	-0.610 (-0.756; -0.465)	-0.330 (-0.481; -0.179)	0.0007	-0.528 (-0.620; -0.435)	-0.338 (-0.430; -0.247)	0.0028
Total GM volume	-1.053 (-1.321; -0.786)	-0.683 (-0.960; -0.407)	0.0138	-0.910 (-1.064; -0.756)	-0.591 (-0.743; -0.439)	0.0025
Total WM volume	-0.181 (-0.373; 0.0118)	-0.140 (-0.339; 0.060)	0.5040	-0.161 (-0.274; -0.048)	-0.091 (-0.203; 0.020)	0.3632
Cortical GM volume	-1.169 (-1.362; -0.976)	-0.737 (-0.936; -0.537)	0.0123	-0.966 (-1.138; -0.795)	-0.631 (-0.800; -0.463)	0.0042
Deep GM volume	-0.911 (-1.093; -0.728)	-0.774 (-0.963; -0.585)	0.1728	-0.793 (-0.918; -0.669)	-0.417 (-0.539; -0.295)	<0.0001
Thalamic volume	-1.777 (-2.041; -1.514)	-1.658 (-1.930; -1.385)	0.3837	-1.476 (-1.652; -1.300)	-0.968 (-1.141; -0.794)	<0.0001
Ventricular system volume	1.149 (0.584; 1.713)	-0.317 (-0.902; 0.268)	0.0002	1.189 (0.819; 1.559)	0.726 (0.360; 1.091)	0.0658
Cerebellar volume	0.026 (-0.215; 0.266)	0.331 (0.082; 0.579)	0.0414	-0.386 (-0.523; -0.248)	-0.402 (-0.538; -0.266)	0.7529
Mean CTh	-0.668 (-0.955; -0.381)	-0.227 (-0.524; 0.070)	0.0237	-0.513 (-0.673; -0.354)	-0.212 (-0.368; -0.056)	0.0058

For both comparisons (PIRA vs Stable and Relapsing vs Stable) annualized percentage changes (APC) were calculated as the estimate of time in the two clinical groups in linear mixed models using: brain measures at each given time point as dependent variables; total intracranial volume (TIV), sex, age at baseline, disease duration at baseline, and the interactions between sex and baseline disease duration with time as fixed-effect covariates; random intercepts (for subjects and MRI protocols), and a random slope (on time). Reported p-values, unadjusted for multiple comparisons, are referred to the interaction term between time and group.
Abbreviations: PIRA, progression independent of relapse activity; APC, annualized percentage change; CI, confidence interval; GM, gray matter; WM, white matter; CTh, cortical thickness.

© 2022 Cagol A et al. *JAMA Neurol*.

eFigure 1: Annualized total brain volume loss in clinical subgroups

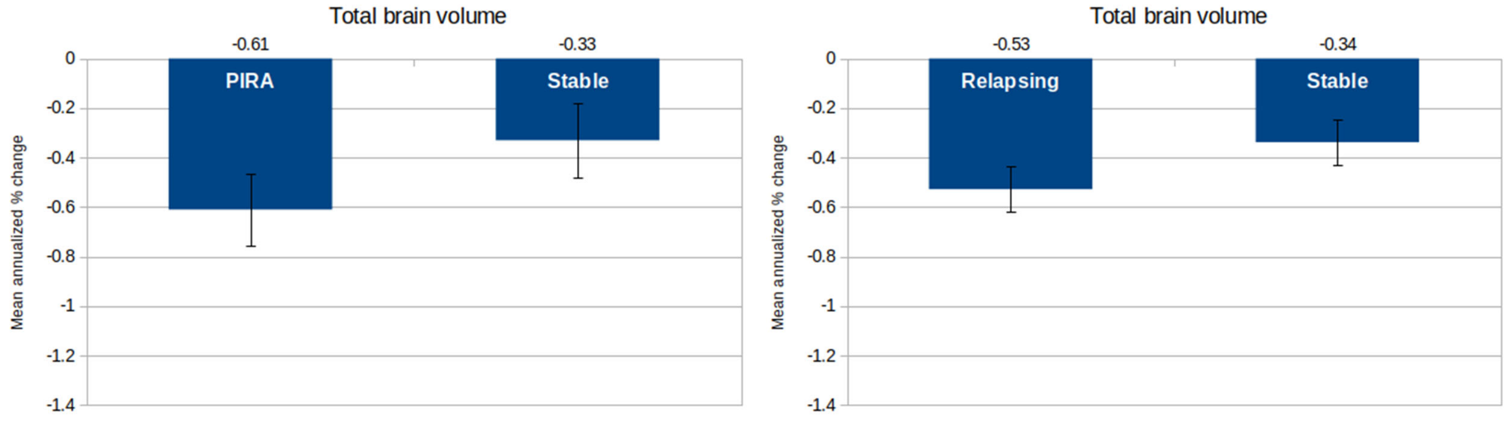

eFigure 2: Annualized total gray matter volume loss in clinical subgroups

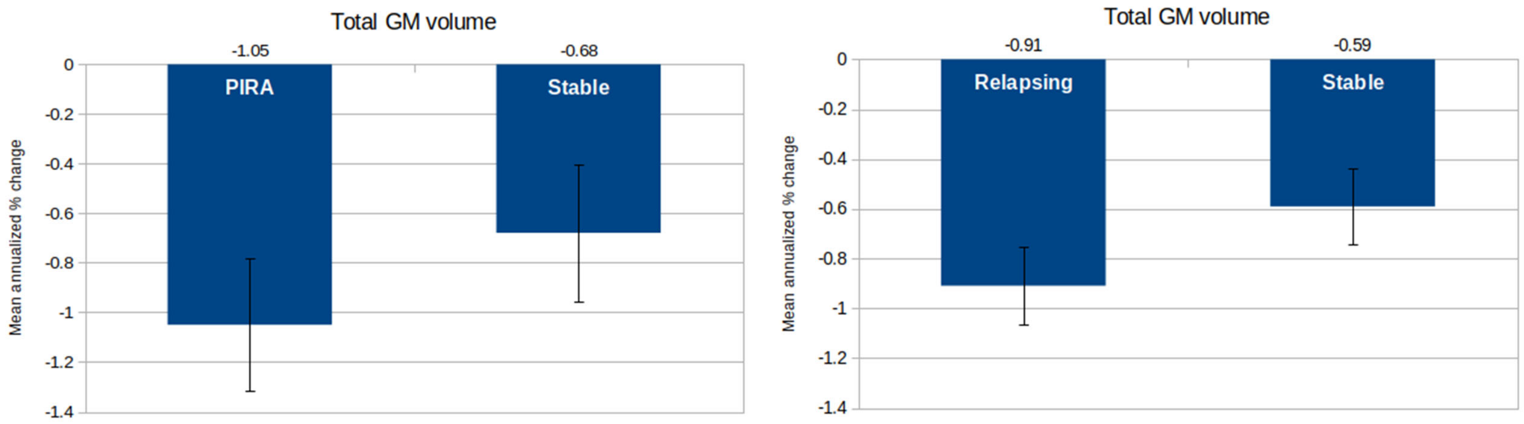

© 2022 Cagol A et al. *JAMA Neurol*.

eMethods

Reliability in brain volumetric measures obtained with different software packages

To support the reliability of brain volumetric measures in our data-set, total brain volume and deep gray matter volume were quantified in all MRI scans also using *SPM12*[1] and *FIRST*,[2] respectively, and brain atrophy rates were obtained also with Structural Image Evaluation, using Normalization, of Atrophy (*SIENA*).[3]

Reliability in brain volumetric measures obtained with different software packages was calculated with the intra-class correlation coefficient (ICC), following a procedure previously proposed for longitudinal MRI studies.[4,5]

The ICC for the estimations of total brain volume obtained with *FreeSurfer* and *SPM12* was 0.88.

The ICC for the estimations of deep gray matter volume obtained with *FreeSurfer* and *FIRST* was 0.91.

The estimations of longitudinal change in total brain volume obtained with *FreeSurfer* were compared to those obtained with *SIENA*. For each subject, the annualized rate of brain volume loss was calculated as the slope of the regression line fitted to all longitudinal changes available for that specific subject, as in De Stefano et al.[6] For subjects where a change in MRI protocol occurred during follow-up, the estimation of brain volume change between the time points immediately before and after MRI protocol change was excluded from the analysis; the overall rate of brain volume loss in such cases was calculated as the mean of the rates before and after protocol change, weighted for the number of time points available in the two epochs.
The ICC in annualized percentage brain volume change between *FreeSurfer* and *SIENA* was 0.72.

Sensitivity analysis: effect of disease-modifying therapies

As a sensitivity analysis, the effect of DMTs on brain atrophy rates was investigated, dividing patients according to the treatment used for the majority of the observation time. DMTs were grouped into three different categories – group 1 (platform DMTs): interferon-beta, and glatiramer-acetate; group 2 (oral DMTs): teriflunomide, dimethyl fumarate, and fingolimod; group 3 (monoclonal antibodies): natalizumab, rituximab, ocrelizumab, and alemtuzumab. Untreated patients were considered as a separate group. The inclusion of treatment group as a covariate in the models investigating the association between explanatory variables and brain atrophy rates did not substantially alter the results.

To evaluate the potential confounding effect of therapeutic switch during observation the comparison of total brain volume loss between (i) patients with PIRA and Stable patients and (ii) Relapsing and Stable patients was performed after excluding (a) patients in which a therapeutic shift between DMTs belonging to different DMTs groups occurred during follow-up; (b) patients in which sphingosine-1-phosphate (S1P) modulators initiation or discontinuation occurred during follow-up.
To preserve matching between groups, for each patient excluded from the analyses the corresponding propensity-score matched subject (belonging to the other group) was excluded.

For the comparison PIRA vs Stable:

- in 6/46 PIRA patients and 9/46 Stable patients there was a switch between DMTs of different groups during follow-up; after excluding such patients from the analysis the mean difference in annualized percentage brain volume change between groups remained significant [MD-APC: -0.448 (95% CI: -0.716; -0.181), p=0.001]
- in 4/46 PIRA patients and 4/46 Stable patients there was a initiation/discontinuation of S1P modulators during follow-up; after excluding such patients from the analysis the mean difference in annualized percentage brain volume change between groups remained significant [MD-APC: -0.547 (95% CI: -0.917; -0.176), p=0.004]

For the comparison Relapsing vs Stable:

- in 43/122 Relapsing patients and 28/122 Stable patients there was a switch between DMTs of different groups during follow-up; after excluding such patients from the analysis the mean difference in annualized percentage brain volume change between groups remained significant [MD-APC: -0.175 (95% CI: -0.328; -0.021), p=0.02]
- in 34/122 Relapsing patients and 10/122 Stable patients there was an initiation/discontinuation of S1P modulators during follow-up; after excluding such patients from the analysis the mean difference in annualized percentage brain volume change between groups remained significant [MD-APC: -0.177 (95% CI: -0.339; -0.016), p=0.03].

© 2022 Cagol A et al. *JAMA Neurol*.

eReferences

1 www.fil.ion.ucl.ac.uk/spm/software/spm12; London, UK

2 FMRIB Integrated Registration and Segmentation Tool, v5.0, Oxford, UK; https://fsl.fmrib.ox.ac.uk/fsl/fslwiki/FIRST

3 Smith SM, Zhang Y, Jenkinson M, et al. Accurate, robust, and automated longitudinal and cross-sectional brain change analysis. Neuroimage. 2002;17(1):479-489. doi:10.1006/nimg.2002.1040

4 Cannon TD, Sun F, McEwen SJ, et al. Reliability of neuroanatomical measurements in a multisite longitudinal study of youth at risk for psychosis. Hum Brain Mapp. 2014;35(5):2424-2434. doi:10.1002/hbm.22338

5 Eshaghi A, Prados F, Brownlee WJ, et al. Deep gray matter volume loss drives disability worsening in multiple sclerosis. Ann Neurol. 2018;83(2):210-222. doi:10.1002/ana.25145.

6 De Stefano N, Stromillo ML, Giorgio A, et al. Establishing pathological cut-offs of brain atrophy rates in multiple sclerosis. J Neurol Neurosurg Psychiatry. 2016;87(1):93-99. doi:10.1136/jnnp-2014-309903

© 2022 Cagol A et al. *JAMA Neurol*.
